# Observation of quantum Darwinism and the origin of classicality with superconducting circuits

**DOI:** 10.1126/sciadv.adx6857

**Published:** 2025-08-01

**Authors:** Zitian Zhu, Kiera Salice, Akram Touil, Zehang Bao, Zixuan Song, Pengfei Zhang, Hekang Li, Zhen Wang, Chao Song, Qiujiang Guo, H. Wang, Rubem Mondaini

**Affiliations:** ^1^ School of Physics, ZJU-Hangzhou Global Scientific and Technological Innovation Center, and Zhejiang Key Laboratory of Micro-nano Quantum Chips and Quantum Control, Zhejiang University, Hangzhou, China.; ^2^ Department of Physics, University of Houston, Houston, TX 77204, USA.; ^3^ Theoretical Division, Los Alamos National Laboratory, Los Alamos, NM 87545, USA.; ^4^ State Key Laboratory for Extreme Photonics and Instrumentation, Zhejiang University, Hangzhou, China.; ^5^ Texas Center for Superconductivity, University of Houston, Houston, TX 77204, USA.

## Abstract

The transition from quantum to classical behavior is a central question in modern physics. How can we rationalize everyday classical observations from an inherently quantum world? Quantum Darwinism offers a compelling framework to explain this by proposing that the environment redundantly encodes information about a quantum system, leading to the objective reality. Here, by leveraging cutting-edge superconducting quantum circuits, we observe the highly structured branching quantum states that support classicality and the saturation of quantum mutual information, establishing a robust verification of quantum Darwinism and the underlying geometric structure of quantum states. Additionally, we propose a particular class of observables that can be used as a computationally and experimentally inexpensive quantifier to probe quantum-to-classical transitions. Our investigation delves into how the quantum effects are inaccessible to observers, allowing only classical properties to be detected. It experimentally demonstrates the physical framework through which everyday classical observations emerge from underlying quantum principles and paves the way to settling the measurement problem.

## Teaser

Branching structure of quantum states supports the emergence of classical reality in everyday life from a quantum universe.

## INTRODUCTION

Quantum mechanics markedly upsets our intuitive understanding of nature, changing the long-held view that classical reality is an independent and objective state that we merely observe. As we near the 100th anniversary of quantum mechanics, the long-standing question of how the classical world emerges from the quantum realm remains one of the most profound challenges in modern physics. Parts of this puzzle are now more evident: It is clear that quantum systems cannot be fully understood in isolation; their interactions with the environment must often be considered, leading to the development of quantum decoherence theory (*1*–*3*). By treating the universe as a collection of interacting quantum systems, one thus considers how the environment monitors certain observables of a system of interest. This monitoring destroys quantum coherences, causing the emergence of a preferred set of stable states (*4*, *5*), dubbed “pointer states” (supplementary text section 1A). The process by which these survive is termed “einselection,” short for environment-induced superselection (*6*).

Decoherence is fundamental because it explains why quantum systems, despite their coherent nature, give rise to classical-like behavior. Namely, it ensures that quantum superpositions turn into classical joint probability distributions localized at specific outcomes, explaining why we never observe macroscopic superpositions in our daily lives. Still, it does not fully answer how classicality is perceived in a quantum universe. For that, quantum Darwinism (*6*–*23*) expands upon decoherence, by asserting that the environment not only causes decoherence but also redundantly encodes classical information about the system’s pointer states across its distinct fragments. This redundancy allows different observers to indirectly and independently access and confirm the classical state of the system without disturbing it. Decoherence, therefore, plays a dual role in quantum-to-classical transitions: Suppressing quantum coherence while ensuring that classical information is robust and accessible in the environment, laying the foundation for the emergence of objective classical reality.

**Fig. 1.**
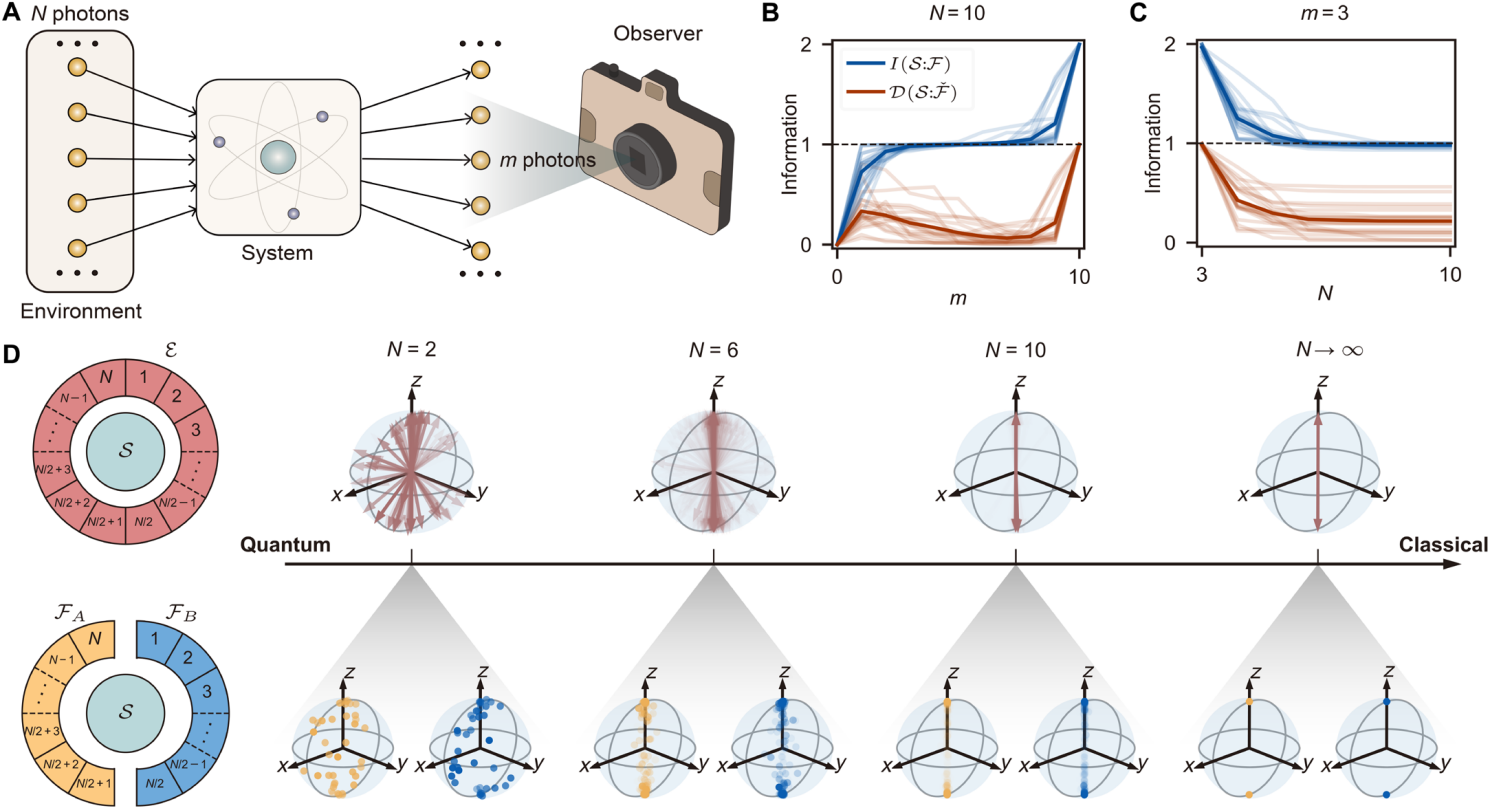
Quantum Darwinism and the emergent classical reality. (**A**) An observer eavesdrops on the system via a fragment of the environment. In macroscopic world, the observer can only access a small fraction (m photons) of N photons as evidence to describe the system. (**B**) Mutual information I(S:F) and quantum discord $D\left(S:\overset{ˇ}{F}\right)$ as a function of the fragment size m. Conditional gates $\left\{U_{⊘}^{k}\right\}$ are randomly sampled to simulate the interactions between S (a qubit) and photons with relevant quantities numerically calculated over 20 runs; faint lines refer to each random realization, and the solid one is their average. A classical plateau of $H_{S}≃1$ is observed for small F. As m→N, $D\left(S:\overset{ˇ}{F}\right)$ suddenly rises, leading to increased I(S:F), which indicates the existence of quantum correlations between the system and the whole environment. (**C**) I(S:F) and $D\left(S:\overset{ˇ}{F}\right)$ as a function of environment size N with fixed fragment size m=3. Only classical information of S can be redundantly recorded in F at large N, signifying the classicality of macroscopic world. (**D**) A branching structure emerges as the quantum-to-classical transition takes place. Top row: The whole environment E is observed (m=N). With growing N, the system experiences increasing decoherence such that, when N→∞, $|\;\psi_{SE}\;\rangle$ is at the branching state, clustering around the pointer states ∣0〉 and ∣1〉 of S. Bottom row: E is separated into two halves $F_{A}$ and $F_{B}$, independently measured by different observers. For small N, e.g., N=2 and 6, observation results strongly depend on the type of interaction due to quantum effects, making them different for various observers. For N→∞, all results of different observers agree, which is what we infer in the classical world.

More specifically, this can be observed as an example from daily life, as illustrated in Fig. 1A, where a camera represents an observer measuring a central system; it can also be equivalently interpreted as taking a picture of an object, say a tree. The camera captures photons scattered from the tree and indirectly learns about the tree’s position through these photons. Now, consider a second observer taking a picture of the tree simultaneously. The photons absorbed by both cameras will differ, but the observers still agree upon the tree’s position, as they have learned the same information. This information can be quantified by the mutual information, $I\left(S:F\right)=H_{S}+H_{F}-H_{SF}$, which is the total bipartite information of the system S and a fragment F of the environment E (total photon bath). Here, $H_{i}=-\text{Tr}\left[\rho_{i}{\text{log}}_{2}\left(\rho_{i}\right)\right]$ is the von Neumann entropy of subsystem i. When all observers learn the same information, a plateau emerges in the mutual information as a function of the fraction of photons captured (Fig. 1B). In general settings, such classical reality only manifests in a large enough environment, i.e., N≫m (Fig. 1C), which is the typical scenario in the macroscopic world. Additionally, the information about purely quantum correlations, known as quantum discord (*24*), $D\left(S:\overset{ˇ}{F}\right)=I\left(S:F\right)-\chi \left(S:\overset{ˇ}{F}\right)$, tends to zero; this is a precise definition of classicality in quantum Darwinism. Here, $\chi \left(S:\overset{ˇ}{F}\right)=H_{S}-{\text{min}}_{\left\{M_{k}^{F}\right\}}\left(H_{S|\overset{ˇ}{F}}\right)$ is the Holevo bound, which quantifies the maximum classical information that one can obtain from an optimal quantum measurement chosen from the set of measurements $\left\{M_{k}^{F}\right\}$ on F, where $H_{S|\overset{ˇ}{F}}$ is the conditional entropy.

The physical mechanism underlying the characterization of classicality can be uncovered by using insights from geometric quantum mechanics (*25*–*27*) alongside the aforementioned information-theoretic quantities. It has been formally shown (*28*) that quantum states tend to cluster around specific classical configurations; such states are formed from the pointer states that survive environmental monitoring and are referred to as branching states (*29*, *30*). In particular, a unique structure of states of the system and environment exists such that local quantum correlations are suppressed, and classical information is redundantly copied in the many information-bearing degrees of freedom of the environment; an example is given in Fig. 1D for the case with system-environment interactions mapped by random quantum unitaries as defined below.

Despite the recent theoretical advancements in delineating the origin of classicality, preliminary experimental results (*31*–*33*) only show limited information-theoretic signatures of quantum Darwinism in special settings, such as encoding redundancy on specific GHZ initial states or realizing observation in a small number of environmental degrees of freedom. In particular, the geometric underpinnings of the global quantum wave function supporting quantum-to-classical transitions are still unexplored. In addition, from an operational perspective, a demonstration that connects the arguments of quantum Darwinism with the practical observing process is still lacking.

Here, we present a comprehensive experimental demonstration of quantum Darwinism and study the self-organizing branching of quantum states through the lens of geometric quantum mechanics. Leveraging the tuning flexibility of our high-quality superconducting quantum qubits (*34*, *35*), which feature energy relaxation time $T_{1}$ around 130 μs, and fidelities of single-qubit gate around 0.9997 and two-qubit controlled-Z (CZ) gate around 0.998 (see supplementary text section 2A), we observe that the formation of classical reality accompanies a clustering around the pointer states of the system’s wave function and further show that the encoded classical information of system can be exactly decoded from environment fragments. In particular, this clustering is a consequence of decoherence, eventually resulting in zero quantum discord. Building upon this insight, we propose an approach for quantifying quantum Darwinism through suitably chosen local observables, facilitating its verification, offering further evidence for the theory, and leading to potential new applications.

## RESULTS

### Branching states and quantum Darwinism

The essence of quantum Darwinism is understanding how the system-environment information exchange leads to the emergence of classicality through encoding copies of the classical information of S in independent fragments of E. The only compatible form of the SE joint state is the singly branching form (*28*), i.e., the one that filters out the system’s pointer states (*29*, *30*). To probe this structure of states, geometric quantum mechanics (*25*–*27*, *36*) emerges as a powerful framework (supplementary text section 1B). Here, the quantum state space is formed by the complex projective Hilbert space $P\left(H\right)=\mathbb{C}P^{D-1}$ for a system with Hilbert space H of dimension D, whose geometric structure is characterized by an invariant measure, the Fubini-Study metric. In particular, a pure state, a point $Z_{0}\in P\left(H\right)$, is represented by a Dirac measure $\mu_{\text{pure}}=\delta_{Z_{0}}$, while a mixed state corresponds to a complex combination of weighted Dirac measures, $\mu_{\text{mix}}=\sum_{j}\lambda_{j}\delta_{Z_{j}}$, with $\sum_{j}\lambda_{j}=1$. Within this picture, any joint pure state of SE, undergoing decoherence, can be expressed as

$$\begin{array}{c} |\psi_{\mathrm{SE}}\rangle =\sum_{i,\alpha ,\beta }\psi_{i\text{αβ}}|s_{i}\rangle |f_{\alpha }\rangle |{\bar{f}}_{\beta }\rangle \\ =\sum_{\alpha ,\beta }\sqrt{X_{\text{αβ}}}|\chi_{\text{αβ}}\rangle |f_{\alpha }\rangle |{\bar{f}}_{\beta }\rangle \end{array}$$ (1)

with $|s_{i}\rangle$, $|f_{\alpha }\rangle$, and $|{\bar{f}}_{\beta }\rangle$ being orthonormal states of S, F, and of the environment complement $\bar{F}$, respectively; $X_{\alpha \beta }$ is the probability of SE in the composite state $|\chi_{\alpha \beta }\rangle |f_{\alpha }\rangle |{\bar{f}}_{\beta }\rangle$. This representation visualizes the state as a measure on the projective Hilbert space $P\left(H_{S}\right)$, such that decoherence manifests as the geometric state of S, $\mu_{S}=\sum_{\alpha ,\beta }X_{\alpha \beta }\delta_{\chi_{\alpha \beta }}$, begins to cluster around the pointer states.

For instance, let a system composed of a single-qubit S interacting with an environment E with N-qubits via the conditional gate (*11*), $U_{⊘}^{k}=|\;0_{S}\;\rangle \langle 0_{S}\;|⊗U_{k}^{0}+|\;1_{S}\;\rangle \langle 1_{S}\;|⊗U_{k}^{1}$, where $|\;0_{S}\;\rangle ,|\;1_{S}\;\rangle$ are two orthogonal pointer states of S, and the controlled unitary to the kth environment qubit, $E_{k}$, is $U_{k}^{j}=\text{exp}\left[\left(-{\mathrm{i\theta}}_{k}^{j}/2\right)\left(\sigma_{x}\text{cos}\phi_{k}^{j}+\sigma_{y}\text{sin}\phi_{k}^{j}\right)\right]$. The randomly chosen parameters $\left\{\;\theta_{k}^{j};\phi_{k}^{j}\;\right\}\in \{\;[\left(j-0.5\right)\pi ,\pi ,\left(j+0.5\right)\pi );$[−π,π)} quantify the imperfect encoding of the information about S in E. Given $|\;0_{S}\;\rangle =|\;0\;\rangle$ and $|\;1_{S}\;\rangle =|\;1\;\rangle$, the form of the unitary leads $\mu_{S}$ to develop two clusters at antipodal points on the Bloch sphere for sufficiently large N (Fig. 1D, top), as a result of decoherence, and indicates the einselection of stable pointer states in the macroscopic “classical world.” If instead the environment is subdivided into two disjoint fragments $F_{A}$ and $F_{B}$, then the geometric quantum states, $\mu_{S}^{A}$ and $\mu_{S}^{B}$, equivalently cluster to two deterministic states at large N (Fig. 1D, bottom), while large uncertainty arises at small N due to the residual quantum coherence. The emergence of classicality now becomes clear: Two independent observers eavesdropping on separate environments agree on the measured information of the system.

**Fig. 2.**
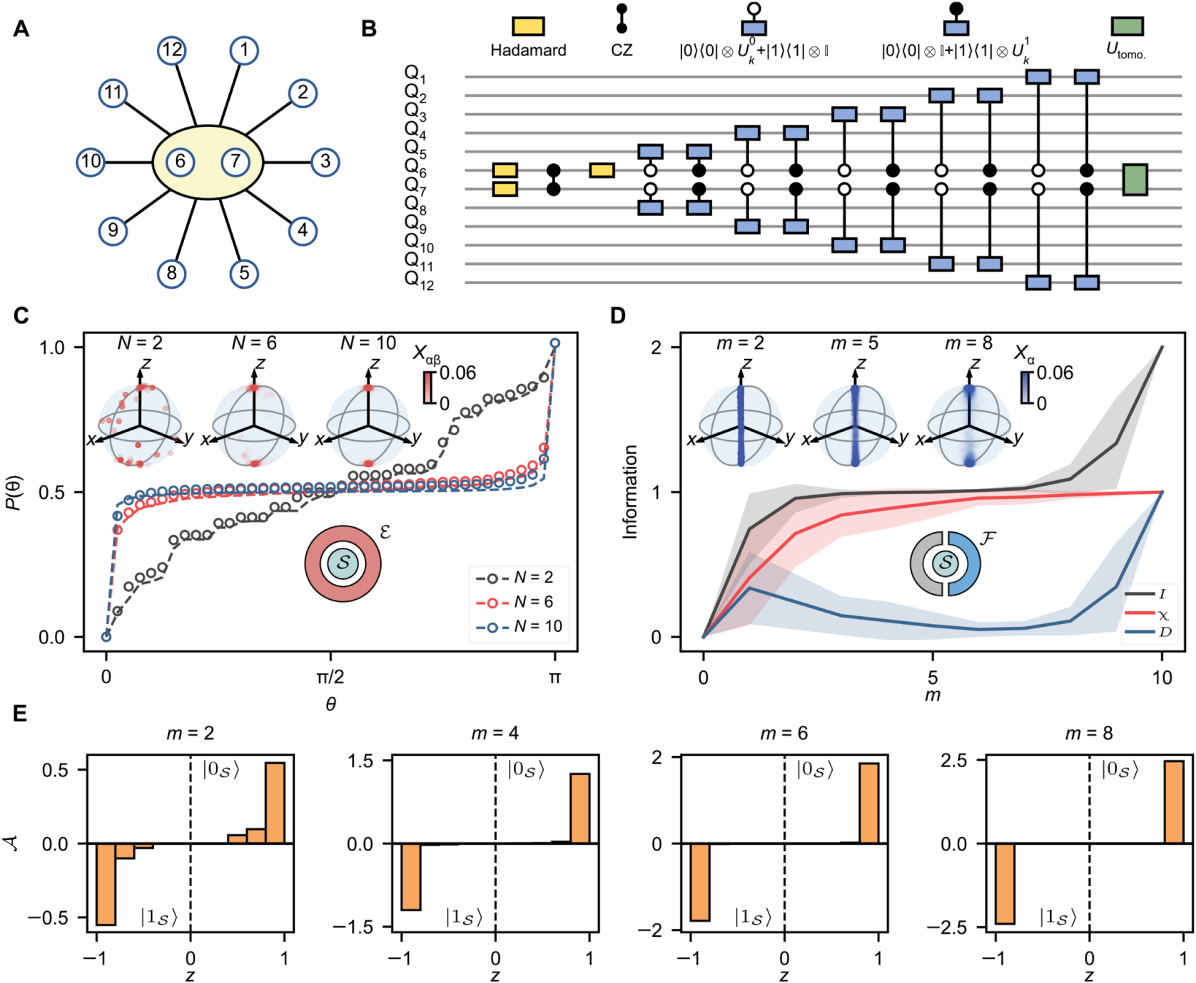
Quantum-to-classical transition: emergence of branching structures. (**A**) A diagram detailing the system-environment interaction topology. We choose two qubits, Q_6_ and Q_7_, as the system and the remaining 10 as the environment. Black lines represent the interaction between the system and the environment. (**B**) Quantum circuit used to simulate the interaction. Similar to Fig. 1, $\theta_{k}^{j}$ and $\phi_{k}^{j}$ in $U_{k}^{j}$ are randomly sampled from the uniform distribution [(j−0.5)π,(j+0.5)π) and [−π,π), respectively. (**C**) Experimental results of the integrated probability P(θ) and the distribution of geometric state $\mu_{S}$ on the Bloch sphere (inset). Black, red, and blue points (dashed lines) represent experimental (noisy simulation, see supplementary text section 2C) results with increasing environment size N. Red dots in the Bloch spheres depict the experimentally reconstructed $\left\{X_{\alpha \beta },\rho_{S}^{\alpha \beta }\right\}$. (**D**) Measured $\left\{X_{\alpha },\rho_{S}^{\alpha }\right\}$ for fragment size m=2,5,and 8 (inset) and numerical results of the mutual information I(S:F), Holevo bound $\chi \left(S:\overset{ˇ}{F}\right)$, and quantum discord $D\left(S:\overset{ˇ}{F}\right)$ as a function of m; the environment size is fixed with N=10. Solid lines are the average values over 10 random realizations where shadows indicate the standard deviations for each quantity. The blue dots in the Bloch spheres (inset) represent the experimentally reconstructed $\left\{X_{\alpha },\rho_{S}^{\alpha }\right\}$. (**E**) Histograms of distributions of A(z) along the *z* axis of the Bloch sphere for fragment size m=2,4,6, and 8. The sign of A(z) builds up a one-to-one correspondence with the pointer states $\left\{\;|\;0_{S}\;\rangle ,|\;1_{S}\;\rangle \;\right\}$.

Moving to experimental exploration, we use 12 qubits on our superconducting processor (supplementary text section 2B) to construct a slightly more complex scheme that comprised a system S formed by two central entangled qubits that are coupled to N=10 surrounding qubits simulating the photon environment E (Fig. 2A); the “photons” only interact with the system and not with each other. Here, a similar randomized conditional gate $U_{⊘}^{k}$ is defined with pointer states $|\;0_{S}\;\rangle =|\;00\;\rangle$ and $|\;1_{S}\;\rangle =|\;11\;\rangle$. In our experiments, S is initialized to $|\Psi_{S}^{0}\rangle =\frac{1}{\sqrt{2}}\left(|00\rangle +|11\rangle \right)$ by applying three Hadamard gates and a CZ gate (Fig. 2B). Subsequent application of conditional gates $\left\{U_{⊘}^{k}\right\}$ correlates all environment qubits $\left\{\;E_{k}\;\right\}$ with S, resulting in the branching state (*9*)

$$|\Psi_{SE}^{⊘}\rangle =\frac{1}{\sqrt{2}}\left(|00\rangle \overset{N}{\underset{k=1}{⊗}}|0_{E_{k}}\rangle +|11\rangle \overset{N}{\underset{k=1}{⊗}}|1_{E_{k}}\rangle \right)\;$$ (2)

where $|\;j_{E_{k}}\;\rangle =\text{cos}\left(\theta_{k}^{j}/2\right)|\;0^{k}\;\rangle -i\text{sin}\left(\theta_{k}^{j}/2\right)e^{i\phi_{k}^{j}}|\;1^{k}\;\rangle$ (j=0,1), recording the information about the pointer states on the kth environment qubit $E_{k}$. See supplementary text section 1C for the analytical calculation of this model.

To experimentally measure the geometric state $\mu_{S}$, we apply quantum state tomography (QST) pulses (see Materials and Methods for details) on S before performing projective measurements on all qubits in the computational basis. Note that the circuit (Fig. 2B) is further compiled into combinations of CZ gates and single-qubit rotations during execution (supplementary text section 2B). From the measurement outcomes, we reconstruct the ensemble realizations $\left\{X_{\alpha \beta },\rho_{S}^{\alpha \beta }\right\}$ of S and record the corresponding basis $|f_{\alpha }\rangle |{\bar{f}}_{\beta }\rangle$ of the N-qubit environment, where $\rho_{S}^{\alpha \beta }$ is the density matrix of $|\;\chi_{\alpha \beta }\;\rangle$. The inset of Fig. 2C visualizes the measured $\left\{X_{\alpha \beta },\rho_{S}^{\alpha \beta }\right\}$ on the Bloch sphere of pointer states for different environment sizes N=2,6,and10. Notably, although the interactions between S and each composition of E are randomly sampled, a branching structure naturally arises through the decoherence induced by the growing size of the environment. When defining the integrated probability P(θ) of the states whose polar angles θ belong to the interval [0,θ], we obtain a direct estimation of the clustering of $\left\{X_{\alpha \beta },\rho_{S}^{\alpha \beta }\right\}$ around the pointer states $|\;\;0_{S}\;\rangle$ and $|\;1_{S}\;\rangle$ (the two poles of the Bloch sphere, Fig. 2C). As the environment size N grows, P(θ) becomes sharper at the two poles while leveling off at 0.5 for intermediate values of the polar angle [θ∈(0,π)]. It is noteworthy that this observed self-organized branching and losing of quantum coherence in our experiments is a pure effect of quantum unitary evolution, without any extra assumptions on measurements, which sheds light on settling the measurement problem, a fundamental postulate of quantum mechanics (*4*, *9*).

Until now, all descriptions have been established in the quantum realm. A natural question then arises: How does the emerging branching structure of the globally pure wave function lead to classical reality? In quantum Darwinism, a key insight about the classical world is that the observer can only eavesdrop on a fragment F of the whole environment E and deduce the information of S from the recorded basis $\left\{\;|f_{\alpha }\rangle \;\right\}$ of F. To bridge the branching behavior and the information-theoretic signatures of quantum Darwinism, we focus on the system with an environment size N=10 and vary the fragment size m of F. Figure 2D displays the numerical results of the mutual information I(S:F), Holevo bound $\chi \left(S:\overset{ˇ}{F}\right)$, and discord $D\left(S:\overset{ˇ}{F}\right)$ and exemplifies the experimentally measured geometric states $\left\{X_{\alpha },\rho_{S}^{\alpha }\right\}$ of S for m=2,5,8 (inset), which are chosen from the classical plateau I(S:F)≃1. Here, $\rho_{S}^{\alpha }=\sum_{\beta }\langle {\bar{f}}_{\beta }\;|\langle f_{\alpha }\;|\;\Psi_{SE}\;\rangle \langle \;\Psi_{SE}\;|\;f_{\alpha }\;\rangle |\;{\bar{f}}_{\beta }\rangle$ is a mixed state due to a lack of information about $\bar{F}$, and $X_{\alpha }$ is the corresponding probability. Within the plateau regime, almost all correlations between S and F are classical, resulting in the observer having the ability to learn most of the shared information between S and F through measurements on F, apart from a measure-zero case where particular measurements reveal no information (*37*). Therefore, $\chi \left(S:\overset{ˇ}{F}\right)≃I\left(S:F\right)$ and $D\left(S:\overset{ˇ}{F}\right)≃0$ at sufficiently large m/N within the plateau. Correspondingly, $\left\{X_{\alpha },\rho_{S}^{\alpha }\right\}$ gradually converge to two clouds around the pointer states $\left\{\;|\;0_{S}\;\rangle ,|\;1_{S}\;\rangle \;\right\}$ with a larger separation along the *z* axis as m grows.

Another angle to show that the state ensemble $\left\{\rho_{S}^{\alpha }\right\}$ of S is classically correlated with the bases $\left\{\;|f_{\alpha }\;\rangle \;\right\}$ ($f_{\alpha }=f_{1}^{\alpha }f_{2}^{\alpha }\ldots f_{m}^{\alpha },f_{i}^{\alpha }\in \left\{\;0,1\;\right\}$) of F that are eavesdropped by the observer is shown in Fig. 2E. Here, we report the measured signal of $A\left(z\right)=\sum_{\left\{\alpha ,\langle \sigma_{z}^{\alpha }\rangle =z\right\}}X_{\alpha }\sum_{i=1}^{m}\left(1-2f_{i}^{\alpha }\right)$ along Bloch sphere’s *z* axis, where $\langle \sigma_{z}^{\alpha }\rangle =\text{Tr}\left(\rho_{S}^{\alpha }\sigma_{z}\right)$. Two branches also emerge in the distribution of A(z), establishing a one-to-one correspondence with the two pointer states of S: $A<0\Rightarrow |\;1_{S}\;\rangle$, $A>0\Rightarrow |\;0_{S}\;\rangle$. Thus, an observer can learn which pointer state the system S “collapses” into by calculating A(z) from the eavesdropped classical bit string $f_{\alpha }$ imprinted on F. As m increases, A(z) tends to congregate to z=±1 with higher signal amplitudes, allowing the observer more confidence to confirm the already known information. Therefore, extra data provided by larger fragments are redundant. As long as the observers eavesdrop on a suitably large F, they always agree on their conclusion if they are in the same branch. These observations illustrate how classical reality emerges from a structured quantum universe and builds up its connection with the classical plateau of mutual information.

### Decoherence and quantum Darwinism

**Fig. 3.**
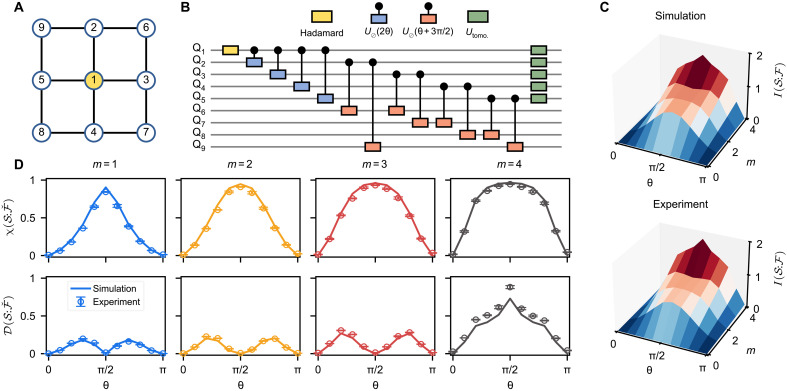
Robustness of the predictions of quantum Darwinism. (**A**) The nine-qubit lattice used in the experiment. Here, Q_1_ is the system, Q_2_-Q_5_ acts as the entangled environment, and Q_6_-Q_9_ serves as the perturbation of the environment. The interaction is realized with conditional gate $U_{⊘}$. (**B**) Schematic of the quantum circuit used to measure the main information theoretic quantities. Initially, Q_1_ is prepared in a superposition of ∣0〉 and ∣1〉 states by applying a Hadamard gate; subsequently, four $U_{⊘}\left(2\theta \right)$ are applied to correlate the system and four environment qubits. Before measuring, the perturbation is added to the environment through the conditional gate $U_{⊘}\left(\theta +3\pi /2\right)$. (**C**) Numerical and experimental results of measuring mutual information between the system and the environment. (**D**) Experimental results of measuring the Holevo bound $\chi \left(S:\overset{ˇ}{F}\right)$ (top) and quantum discord $D\left(S:\overset{ˇ}{F}\right)$ (bottom). The markers (lines) are the experimental (numerical) results. Data points are measured over five independent runs, and error bars represent the standard deviations of these results.

To fully comprehend and test the quantum-to-classical transitions, we now measure the information-theoretic signatures of classicality in quantum Darwinism, i.e., the plateau of mutual information and the vanishing discord. For that, we now use a slightly smaller circuit featuring nine qubits (Fig. 3A), with a single qubit in S. At the same time, we investigate a more generic quantum system where four environment qubits interact weakly via an extra set of four auxiliary ones, allowing for the verification of the emergence of classicality with the interplay of information scrambling in E (*22*, *38*, *39*). The couplings between S and the four directly connected environment qubits are homogeneous and realized via a conditional gate $U_{⊘}\left(\theta \right)=|0_{S}\rangle \langle 0_{S}|⊗𝕀+|1_{S}\rangle \langle 1_{S}|⊗\text{exp}\left[-{i\theta \sigma }_{y}/2\right]$, where the pointer states $|0_{S}\rangle =|0\rangle ,|1_{S}\rangle =|1\rangle$.

Similar to previous cases, the system S starts in a pure state $|\Psi_{S}^{0}\rangle =\frac{1}{\sqrt{2}}\left(|0\rangle +|1\rangle \right)$ by applying a Hadamard gate, and the unitary $U_{⊘}\left(2\theta \right)$ correlates each qubit in E with S, resulting on a branching state

$$|\Psi_{\mathrm{SE}}^{⊘}\rangle =\frac{1}{\sqrt{2}}\;\left(|0\rangle \overset{N}{\underset{k=1}{⊗}}|0_{E_{k}}\rangle +|1\rangle \overset{N}{\underset{k=1}{⊗}}|1_{E_{k}}\rangle \right)\;$$ (3)

In addition, we couple the four environment qubits through four auxiliary qubits with a similar unitary but different interaction “strength” $U_{⊘}\left(\theta +3\pi /2\right)$. The full quantum circuit is shown in Fig. 3B. By varying θ∈[0,π], we experimentally measure the mutual information I(S:F) (Fig. 3C), the Holevo bound $\chi \left(S:\overset{ˇ}{F}\right)$ (Fig. 3D), and the quantum discord $D\left(S:\overset{ˇ}{F}\right)$ (Fig. 3D, see Materials and Methods for measurement details) using full QST for a single initialized state in the nine-qubit lattice (Fig. 3A). The experimental findings in Fig. 3 (C and D) accomplish complete verification of the predictions of quantum Darwinism.

In particular, we observe that, when one expects the “classical” limit to set in (i.e., for θ∈[π/2−ε,π/2+ε] where the extra environment scrambling unitaries amount to identity operations, thus decoupled), the mutual information has a steep rise to the classical plateau region where $I\left(S:F\right)≃H_{S}$ for environment fragment size m=1. Capturing more qubits does not change its value unless we include almost all of the environment in the fragment (m=4), in which case $I\left(S:F\right)\to 2H_{S}$. The experimentally measured I(S:F) for m=4 at θ=π/2 is about 1.83. Additionally, in the same regime, the quantum discord is arbitrarily close to zero until we capture the whole environment, in which case we obtain a peak where $D\left(S:\overset{ˇ}{F}\right)\to H_{S}$ for m=N=4 and θ=π/2, indicating that quantum correlations are a global property of the composite system. This directly confirms that the emergence of classicality is quite robust, even if small imperfections exist, e.g., weak environmental couplings.

### Local observables

Despite the success of probing branching structures and information-theoretic measures to interpret the origin of classical reality, obtaining them for large systems is experimentally and numerically prohibitive due to unscalable quantum tomography and matrix diagonalization. However, the insight of a highly structured quantum wave function provided by geometric quantum mechanics inspires us to propose a new quantifier O to witness quantum Darwinism. O is given by

O=A⊗B⊗I⊗⋯⊗I (4)

where A acts on the system S and B acts on a fragment F of the environment. We note that O is local (i.e., a few-body operator) with a minimal structure, which we will argue to be able to build a one-to-one correspondence between the vanishing of its expectation value and the emergence of a branching structure. For example, if the system exhibits the set of pointer states {∣n〉}, then one can choose A such that it rotates a pointer state to an orthogonal one (e.g., A∣n〉→∣n+1〉). Additionally, we must choose an operator B such that 〈O〉≠0 when the composite state $|\;\psi_{SE}\;\rangle$ is not in a branching structure. This minimal form of O is necessary because observables acting solely on the system may not capture the essential dynamics associated with the branching structures. As a result of this form, 〈O〉 becomes arbitrarily close to zero as long as the system approaches a branching form. This behavior provides a direct and inexpensive method to detect the emergence of a branching structure and the plateau characteristic of quantum Darwinism. In supplementary text section 1D, we provide analytical proof of the behavior of such local observables and further argue for the necessity of the existence of B.

**Fig. 4.**
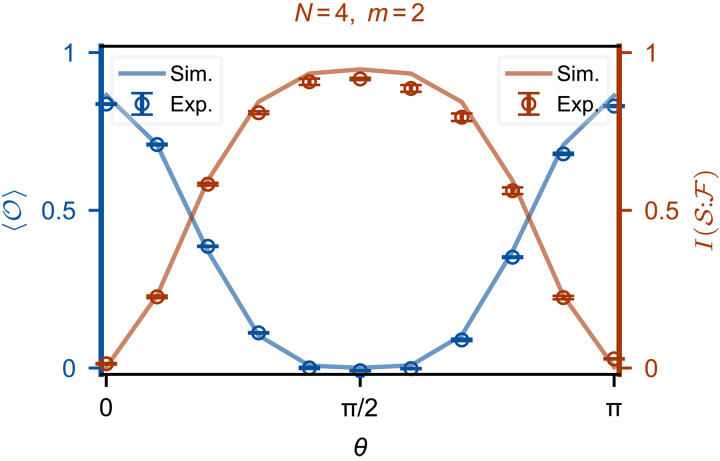
Witnessing quantum Darwinism with local observables. The expectation value of $O=\sigma_{x}⊗\frac{1}{\sqrt{5}}\left(2\sigma_{z}+\sigma_{y}\right)⊗𝕀⊗𝕀\cdots 𝕀$ and mutual information I(S:F) as a function of θ∈[0,π]. The circles (solid lines) represent experimental (numerical) results. The plateau at zero is established when θ is close to π/2. The mutual information between two environment qubits and the system averaged over all combinations for m=2, N=4. Measuring I(S:F) is time-consuming as full QST is used. Data points are measured over five runs, and error bars represent the standard deviations of these five results.

Turning to the experiments, for the circuit in Fig. 3B, instead of tomography, we directly measure the expectation of the observable $O=\sigma_{x}⊗\left(1/\sqrt{5}\right)\left(2\sigma_{z}+\sigma_{y}\right)⊗𝕀⊗𝕀\cdots 𝕀$, see Fig. 4. Here, 〈O〉 is measured for θ between 0 and π for an environmental size N=4; in comparison, the mutual information I(S:F) is measured for a fragment size m=2. We observe a one-to-one correspondence with the zero plateau of 〈O〉 and the emergence of the branching structure as captured by the plateau of I(S:F), demonstrating a convenient and inexpensive way to detect the emergent classical reality.

## DISCUSSION

Understanding the principles of quantum mechanics remains a substantial challenge in physics due to the inherently nonintuitive nature of quantum phenomena, including that of the measurement problem. Here, we presented a robust experimental verification of the predictions of quantum Darwinism (*16*, *29*, *40*–*45*), a physical framework that has the merit of addressing such foundational divide between quantum and classical worlds. At its core is the formation of a branching structure of the global state promoted by the decoherence of a system of interest under the action of its witnessing environment. The tools of geometric quantum mechanics allow the direct observation of branching and the resulting clustering around the system’s pointer states, which supports the emergence of classical reality.

Addressing previous limitations of experimental efforts, our investigation further shows that carefully chosen yet simple quantifiers, such as certain local observables, can be used for probing quantum-to-classical transitions. With quantum Darwinism now experimentally validated and established as a mature field of research, we can explore its rich potential in addressing further issues of the quantum measurement problem, such as the dynamics of the collapse of the wave function. One potential avenue is studying the interplay between the emergence of classicality and thermalization in open quantum systems. Our findings thus pave the way for connecting two of the most successful fields in physics: thermodynamics and quantum theory.

## MATERIALS AND METHODS

### Quantum state tomography

To measure the information-theoretic quantities, such as mutual information and quantum discord, we need the density matrices of the system S and environment E, which can be obtained by performing QST (*46*). To this end, we apply ${\left\{I,R_{x}\left(\pi /2\right),R_{y}\left(\pi /2\right)\right\}}^{⊗n}$ gates to the related qubits to rotate the measurement basis; this means that one needs to repeat the circuit 3*^n^* times, where n is the number of qubits. Therefore, in Figs. 2B and 3B, the circuits end up with tomography gates in ${\left\{I,R_{x}\left(\pi /2\right),R_{y}\left(\pi /2\right)\right\}}^{⊗n}$ before measurements. With these measurement results, an overdetermined equation can be solved to reconstruct the density matrix. In practice, the qubit number n varies from 1 to 5. To reduce the measurement time, we only measure n=5 and trace out some qubits to get density matrices for n<5. Note that QST is unscalable for experiments because an exponentially increasing number of tomography operations are needed.

### Measurement of the geometric state

We have shown that any composite state of the system SE can be written as Eq. 1. For the 12-qubit lattice, we consider the two central qubits as the system, whose pointer states are described by logical states $|\;0_{S}\;\rangle =|\;0_{L}\;\rangle =|\;00\;\rangle$ and $|\;1_{S}\;\rangle =|\;1_{L}\;\rangle =|\;11\;\rangle$. Each environment basis $|\;f_{\alpha }\;\rangle |\;{\bar{f}}_{\beta }\;\rangle$ encodes a system state $|\;\chi_{\alpha \beta }\;\rangle =\sqrt{\frac{X_{0,\alpha \beta }}{X_{\alpha \beta }}}|\;0_{L}\;\rangle +\sqrt{\frac{X_{1,\alpha \beta }}{X_{\alpha \beta }}}|\;1_{L}\;\rangle$ with a probability $X_{\alpha \beta }$. To obtain the system state $|\;\chi_{\alpha \beta }\;\rangle$ and the corresponding environment basis $|f_{\alpha }\;\rangle |{\bar{f}}_{\beta }\;\rangle$, we apply logical QST gates

$$I_{L}=I⊗I$$ (5)

$${\mathrm{Rx}}_{L}\left(\pi /2\right)=\text{cos}\left(\pi /4\right)I⊗I-i\text{sin}\left(\pi /4\right)X⊗X$$ (6)

$${\mathrm{Ry}}_{L}\left(\pi /2\right)=\text{cos}\left(\pi /4\right)I⊗I-i\text{sin}\left(\pi /4\right)Y⊗Y$$ (7)

to the central qubits and simultaneously record the measurement outcomes of the environment basis $|f_{\alpha }\;\rangle |{\bar{f}}_{\beta }\;\rangle$. In our experiments, we only keep the measurement outcomes that are in the logical space by postselection, while those results that leak out of the logical basis due to experimental imperfections, e.g., gate errors, are discarded. Then, the experimental $\rho_{\alpha \beta }$ of $|\;\psi_{\alpha \beta }\;\rangle$ can be extracted by QST. The state $\rho_{\alpha \beta }$ can then be visualized on the Bloch sphere with coordinates calculated by $\left[\text{Tr}\left(\rho_{\alpha \beta }\sigma_{x}\right),\text{Tr}\left(\rho_{\alpha \beta }\sigma_{y}\right),\text{Tr}\left(\rho_{\alpha \beta }\sigma_{z}\right)\right]$. In the experimental realization, ${\mathrm{Rx}}_{L}\left(\pi /2\right)$ and ${\mathrm{Ry}}_{L}\left(\pi /2\right)$ are further decomposed into single-qubit rotations and CZ gates, where the matrix form of CZ gate is

$$U_{\text{CZ}}=\left[\begin{array}{cccc} 1 & 0 & 0 & 0\\ 0 & 1 & 0 & 0\\ 0 & 0 & 1 & 0\\ 0 & 0 & 0 & -1 \end{array}\right]$$ (8)

### Measurement of the quantum discord

In Fig. 3, we obtain the density matrix of the central qubit and four environment qubits $\rho_{SF}$ by QST. Here, we introduce how we calculate the quantum discord with $\rho_{SF}$. Quantum discord is defined as (*6*, *24*)

$$D\left(S:\overset{ˇ}{F}\right)=I\left(S:F\right)-\chi \left(S:\overset{ˇ}{F}\right)$$ (9)

We can obtain mutual information I(S:F) straightforwardly for m= 1, 2, 3, and 4 by calculating the von Neumann entropy of the system and environment

$$I\left(S:F\right)=H_{S}+H_{F}-H_{SF}$$ (10)

However, the Holevo bound $\chi \left(S:\overset{ˇ}{F}\right)$ requires calculating the conditional entropy

$$H_{S|\overset{ˇ}{F}}=\underset{\alpha }{\sum p_{\alpha }}H_{S\|f_{\alpha }\rangle }$$ (11)

where $p_{\alpha }=\text{Tr}[\rho_{SF}(I⊗|f_{\alpha }\;\rangle \langle f_{\alpha }\;|)]$ is the probability that F is at state $|f_{\alpha }\;\rangle$. Then, we can calculate the conditional density matrix as

$$\rho_{S\|f_{\alpha }\rangle }={\text{Tr}}_{F}\left[\frac{M_{\alpha }\rho_{\mathrm{SF}}M_{\alpha }^{†}}{\text{Tr}\left(M_{\alpha }\rho_{\mathrm{SF}}M_{\alpha }^{†}\right)}\right]$$ (12)

where $M_{\alpha }=|f_{\alpha }\;\rangle \langle f_{\alpha }\;|$.

## Supplementary Materials

### This PDF file includes:

Supplementary Text Figs. S1 and S2 Table S1 References
