# Supplementary material for: Observation of quantum Darwinism and the origin of classicality with superconducting circuits
Source: Sci Adv. 2025 Aug 1;11(31):eadx6857. doi: 10.1126/sciadv.adx6857 (PMC12315987; doi:10.1126/sciadv.adx6857)
Supplement: Supplementary file 1 — Supplementary Text Figs. S1 and S2 Table S1 References [file sciadv.adx6857_sm.pdf]

Science Advances
AAAS

Supplementary Materials for

Observation of quantum Darwinism and the origin of classicality with superconducting circuits

Zitian Zhu *et al.*

Corresponding author: Qiujiang Guo, qguo@zju.edu.cn; Rubem Mondaini, rmondaini@uh.edu

Sci. Adv. **11**, eadx6857 (2025)
DOI: 10.1126/sciadv.adx6857

This PDF file includes:

Supplementary Text
Figs. S1 and S2
Table S1
References

Supplementary Text 1 - Theoretical Analysis

A. Decoherence theory and pointer states

Decoherence theory addresses how quantum systems interacting with their environment effectively lose their quantum coherence, leading to the destruction of quantum superpositions. When a quantum system $\mathcal{S}$ interacts with its environment $\mathcal{E}$, the total system can be described by a Hamiltonian,

$$H = H_{\text{sys.}} + H_{\text{env.}} + H_{\text{int.}}, \tag{S1}$$

where $H_{\text{sys.}}$ and $H_{\text{env.}}$ are the self-Hamiltonians of the system and environment, respectively, and $H_{\text{int.}}$ represents their interaction. The unitary evolution under this interacting Hamiltonian suffices to build up quantum entanglement over $\mathcal{SE}$, leading to non-separable correlations between $\mathcal{S}$ and $\mathcal{E}$. In this scenario, only a set of preferred states, called pointer states $\{|n_{\mathcal{S}}\rangle\}_n$, are einselected, remaining unchanged under the system-environment interactions [4, 5], while other states are decohered into mixtures of them. Specifically, if an observable to be measured commutes with the total Hamiltonian H such that

$$[H, O_{\mathcal{S}} \otimes \mathbb{I}_{\mathcal{E}}] = 0, \tag{S2}$$

this observable is conserved under the dynamics governed by H and will not be perturbed by the system-environment interaction. In the representation of pointer states, the off-diagonal elements (quantum coherences) of the system's reduced density matrix decay rapidly in the limit of good decoherence, effectively suppressing quantum interference effects. In particular, after decoherence, we obtain,

$$\rho_{\mathcal{S}} = \text{Tr}_{\mathcal{E}}(\rho_{\mathcal{SE}}) = \sum_n p_n |n_{\mathcal{S}}\rangle\langle n_{\mathcal{S}}|, \tag{S3}$$

$$\langle O_{\mathcal{S}}\rangle = \text{Tr}(\rho_{\mathcal{S}} O_{\mathcal{S}}) = \sum_n p_n \langle n_{\mathcal{S}}|O_{\mathcal{S}}|n_{\mathcal{S}}\rangle\ . \tag{S4}$$

Another method to determine the pointer states, known as the 'predictability sieve' [47, 48], involves selecting states that minimize the entropy production due to decoherence, effectively picking states that retain maximal predictability over time. Mathematically, pointer states $\{|n_{\mathcal{S}}\rangle\}_n$ are the states whose von Neumann entropy $H(\rho_{\mathcal{S}}) = -\text{Tr}[\rho_{\mathcal{S}} \log_2(\rho_{\mathcal{S}})]$ is constant over time. When the self-Hamiltonian of the system does not commute with the interaction Hamiltonian the notion of pointer states can be only approximate and in such cases we either look for observables that almost commute with the Hamiltonian $[H, O_{\mathcal{S}} \otimes \mathbb{I}_{\mathcal{E}}] \approx 0$, or the states that have minimal changes in their von Neumann entropy as a function of time.

B. Geometric quantum mechanics

As detailed in Ref. [27, 28], Geometric Quantum Mechanics provides a formal framework for representing quantum states in the complex projective Hilbert space, defined as $\mathcal{P}(\mathcal{H}) = \mathbb{C}P^{D-1}$, instead of the conventional Hilbert space representation for a system with dimension D. This geometric framework has proven instrumental in developing intuition about quantum many-body dynamics. In contrast to the description of density matrices, geometric quantum states are capable of expressing the specific realization of a mixed-state ensemble. More specifically, based on a chosen set of orthonormal basis $\{|e_\alpha\rangle\}_\alpha$ of the Hilbert space, a pure state can be expressed in terms of D complex coordinates as $|Z\rangle = \sum_\alpha Z^\alpha |e_\alpha\rangle$, with the equivalence relation $Z \sim \lambda Z$ for all $\lambda \in \mathbb{C} \setminus \{0\}$ ensuring $Z \in \mathcal{P}(\mathcal{H})$. The state space $\mathcal{P}(\mathcal{H})$ is equipped with differential-geometric tools such as the Fubini-Study volume element. This preferred invariant measure facilitates the use of measure theory for defining ensembles and describing mixed states. Parameterizing pure states using probability-phase coordinates $Z^\alpha = \sqrt{p_\alpha} e^{i\nu_\alpha}$, the Fubini-Study volume element is expressed as $dV_{FS} \sim \prod_{\alpha=0}^{D-1} dp_\alpha\, d\nu_\alpha$, up to an overall normalization factor. The Fubini-Study volume element naturally defines a geometric quantum state as a probability measure μ on $\mathcal{P}(\mathcal{H})$, representing an ensemble of pure states. As we detail in the main text, a pure state $Z_0 \in \mathcal{P}(\mathcal{H})$ corresponds to a Dirac measure $\mu_{\text{pure}} = \delta_{Z_0}$, while a finite ensemble is represented as a convex combination of weighted Dirac measures: $\mu_{\text{ensemble}} = \sum_j \lambda_j \delta_{Z_j}$, with $\sum_j \lambda_j = 1$. The connection to density matrices then arises naturally: The elements $\rho_{\alpha\beta}$ of the density matrix are the expectation values of $Z^\alpha \bar{Z}^\beta$ with respect to μ, given by $\rho_{\alpha\beta} = \mathbb{E}_\mu[Z^\alpha \bar{Z}^\beta] = \int_{\mathcal{P}(\mathcal{H})} d\mu(Z) Z^\alpha \bar{Z}^\beta$. In Ref. [25, 28], this approach was used to derive the geometric quantum state of an open quantum system coupled with an environment. We use this framework to experimentally probe the geometric states of the system of interest $\mathcal{S}$, and verify how the emergence of

classicality reflects these states' structure. More precisely, the joint pure state of the system and environment $|\psi_{\mathcal{SE}}\rangle$ can be represented as

$$\begin{aligned} |\psi_{\mathcal{SE}}\rangle \equiv |\psi_{\mathcal{SF\bar{F}}}\rangle &= \sum_{i,\alpha,\beta} \psi_{i\alpha\beta} \, |s_i\rangle \, |f_\alpha\rangle \, |\bar{f}_\beta\rangle \, , \\ &= \sum_{\alpha,\beta} \sqrt{X_{\alpha\beta}} \, |\chi_{\alpha\beta}\rangle \, |f_\alpha\rangle \, |\bar{f}_\beta\rangle \, , \end{aligned} \tag{S5}$$

which can be interpreted as a measure on the projective Hilbert space $\mathcal{P}(\mathcal{H}_\mathcal{S})$. Decoherence then leads to clustering of the states $|\chi_{\alpha\beta}\rangle$ around pointer states. For instance, in a system comprised of a single qubit with pointer states $|0\rangle$ and $|1\rangle$, this clustering results in geometric quantum states forming two distinct clusters at antipodal points on the Bloch sphere, which is corroborated by the experimental results presented in the main manuscript.

C. Analytical calculation of the mutual information

We provide an in-depth calculation of the mutual information, $I(\mathcal{S}:\mathcal{F}) = H_\mathcal{S} + H_\mathcal{F} - H_{\mathcal{SF}}$, for the system-environment interaction model used in Fig. 2 of the main text, where the system $\mathcal{S}$ interacts with an N-qubit environment $\mathcal{E}$ via conditional gates, $U^k_\oslash = |0_\mathcal{S}\rangle\langle 0_\mathcal{S}| \otimes U^0_k + |1_\mathcal{S}\rangle\langle 1_\mathcal{S}| \otimes U^1_k$, to show that it has a closed expression as studied in Ref. [11]. We will be interested in the correlations between the fragment $\mathcal{F}$ with a size m and the system $\mathcal{S}$. The system starts in the state superposition $|\Psi^0_\mathcal{S}\rangle = \sqrt{p}|0_\mathcal{S}\rangle + \sqrt{q}|1_\mathcal{S}\rangle$, where the normalization coefficients p and q ($p = q = 1/2$ in the main text) satisfy the relation $p + q = 1$, while the environment starts in the ground state $|00...0\rangle$. In the main text, we show that without considering auxiliary qubits, the resulting branching state is

$$|\Psi^\oslash_{\mathcal{SE}}\rangle = \sqrt{p}|0_\mathcal{S}\rangle \bigotimes_{k=1}^{N} |0_{\mathcal{E}_k}\rangle + \sqrt{q}|1_\mathcal{S}\rangle \bigotimes_{k=1}^{N} |1_{\mathcal{E}_k}\rangle, \tag{S6}$$

where $|j_{\mathcal{E}_k}\rangle = \cos(\theta^j_k/2)|0^k\rangle - \mathrm{i}\sin(\theta^j_k/2)e^{\mathrm{i}\phi^j_k}|1^k\rangle$ ($j = 0, 1$). $\{|j_{\mathcal{E}_k}\rangle\}$ composes a set of non-orthogonal basis. Hence the overlap between $|0_{\mathcal{E}_k}\rangle$ and $|1_{\mathcal{E}_k}\rangle$ is

$$s_k = \langle 1_{\mathcal{E}_k}|0_{\mathcal{E}_k}\rangle = \cos(\theta^1_k/2)\cos(\theta^0_k/2) + e^{i(\phi^0_k - \phi^1_k)}\sin(\theta^1_k/2)\sin(\theta^0_k/2). \tag{S7}$$

We can define

$$\begin{aligned} |\mathcal{F}_0\rangle = \bigotimes_{k=1}^{m} |0_{\mathcal{E}_k}\rangle \text{ and } |\bar{\mathcal{F}}_0\rangle = \bigotimes_{k=m+1}^{N} |0_{\mathcal{E}_k}\rangle, \\ |\mathcal{F}_1\rangle = \bigotimes_{k=1}^{m} |1_{\mathcal{E}_k}\rangle \text{ and } |\bar{\mathcal{F}}_1\rangle = \bigotimes_{k=m+1}^{N} |1_{\mathcal{E}_k}\rangle. \end{aligned} \tag{S8}$$

Now, we can express the wave function as

$$\begin{aligned} |\Psi^\oslash_{\mathcal{SE}}\rangle &= \sqrt{p}|0_\mathcal{S}\rangle|\mathcal{F}_0\rangle|\bar{\mathcal{F}}_0\rangle + \sqrt{q}|1_\mathcal{S}\rangle|\mathcal{F}_1\rangle|\bar{\mathcal{F}}_1\rangle \\ &= \sqrt{p}|0_\mathcal{S}\rangle|\mathcal{E}_0\rangle + \sqrt{q}|1_\mathcal{S}\rangle|\mathcal{E}_1\rangle, \end{aligned} \tag{S9}$$

and the overlaps as

$$\langle \mathcal{E}_1|\mathcal{E}_0\rangle = \prod_{k=1}^{N} s_k, \tag{S10}$$

$$\langle \mathcal{F}_1|\mathcal{F}_0\rangle = \prod_{k=1}^{m} s_k, \tag{S11}$$

$$\text{and } \langle \bar{\mathcal{F}}_1|\bar{\mathcal{F}}_0\rangle = \prod_{k=m+1}^{N} s_k. \tag{S12}$$

We then see that the density matrix $\rho_{\mathcal{S}}$ is

$$
\begin{aligned}
\rho_{\mathcal{S}} =& \mathrm{Tr}_{\mathcal{E}}(|\Psi^{\oslash}_{\mathcal{SE}}\rangle\langle\Psi^{\oslash}_{\mathcal{SE}}|) \\
=& \sum_i \langle i| \left[p|0_{\mathcal{S}}\rangle\langle 0_{\mathcal{S}}| \bigotimes |\mathcal{E}_0\rangle\langle\mathcal{E}_0| + q|1_{\mathcal{S}}\rangle\langle 1_{\mathcal{S}}| \bigotimes |\mathcal{E}_1\rangle\langle\mathcal{E}_1| + \sqrt{pq}|0_{\mathcal{S}}\rangle\langle 1_{\mathcal{S}}| \bigotimes |\mathcal{E}_0\rangle\langle\mathcal{E}_1| + \sqrt{pq}|1_{\mathcal{S}}\rangle\langle 0_{\mathcal{S}}| \bigotimes |\mathcal{E}_1\rangle\langle\mathcal{E}_0|\right] |i\rangle \\
=& \sum_i \left[p|0_{\mathcal{S}}\rangle\langle 0_{\mathcal{S}}|\langle i|\mathcal{E}_0\rangle\langle\mathcal{E}_0|i\rangle + q|1_{\mathcal{S}}\rangle\langle 1_{\mathcal{S}}|\langle i|\mathcal{E}_1\rangle\langle\mathcal{E}_1|i\rangle + \sqrt{pq}|0_{\mathcal{S}}\rangle\langle 1_{\mathcal{S}}|\langle i|\mathcal{E}_0\rangle\langle\mathcal{E}_1|i\rangle + \sqrt{pq}|1_{\mathcal{S}}\rangle\langle 0_{\mathcal{S}}|\langle i|\mathcal{E}_1\rangle\langle\mathcal{E}_0|i\rangle\right] \\
=& p|0_{\mathcal{S}}\rangle\langle 0_{\mathcal{S}}| \left(\sum_i \langle\mathcal{E}_0|i\rangle\langle i|\mathcal{E}_0\rangle\right) + q|1_{\mathcal{S}}\rangle\langle 1_{\mathcal{S}}| \left(\sum_i \langle\mathcal{E}_1|i\rangle\langle i|\mathcal{E}_1\rangle\right) \\
&+ \sqrt{pq}|0_{\mathcal{S}}\rangle\langle 1_{\mathcal{S}}| \left(\sum_i \langle\mathcal{E}_1|i\rangle\langle i|\mathcal{E}_0\rangle\right) + \sqrt{pq}|1_{\mathcal{S}}\rangle\langle 0_{\mathcal{S}}| \left(\sum_i \langle\mathcal{E}_0|i\rangle\langle i|\mathcal{E}_1\rangle\right),
\end{aligned}
\tag{S13}
$$

where $|i\rangle \in \{\bigotimes_{k=1}^{N} |j^k\rangle, j = 0, 1\}$. Since the basis set $\{|i\rangle\}$ is orthonormal satisfying $\sum_i |i\rangle\langle i| = \mathbb{I}$, we get

$$
\begin{aligned}
\rho_{\mathcal{S}} &= p|0_{\mathcal{S}}\rangle\langle 0_{\mathcal{S}}| + q|1_{\mathcal{S}}\rangle\langle 1_{\mathcal{S}}| + \sqrt{pq}|0_{\mathcal{S}}\rangle\langle 1_{\mathcal{S}}|\langle\mathcal{E}_1|\mathcal{E}_0\rangle + \sqrt{pq}|1_{\mathcal{S}}\rangle\langle 0_{\mathcal{S}}|\langle\mathcal{E}_0|\mathcal{E}_1\rangle \\
&= \begin{bmatrix} p & \sqrt{pq}\langle\mathcal{E}_1|\mathcal{E}_0\rangle \\ \sqrt{pq}\langle\mathcal{E}_0|\mathcal{E}_1\rangle & q \end{bmatrix} \\
&= \begin{bmatrix} p & \sqrt{pq}\prod_{k=1}^{N} s_k \\ \sqrt{pq}\prod_{k=1}^{N} s_k^* & q \end{bmatrix}.
\end{aligned}
\tag{S14}
$$

Now, the density matrix $\rho_{\mathcal{SF}}$ of the composite system $\mathcal{SF}$ directly follows Eq. (S14) as

$$
\rho_{\mathcal{SF}} = \begin{bmatrix} p & \sqrt{pq}\prod_{k=m+1}^{N} s_k \\ \sqrt{pq}\prod_{k=m+1}^{N} s_k^* & q \end{bmatrix}.
\tag{S15}
$$

Similarly, we get

$$
\rho_{\mathcal{S}\bar{\mathcal{F}}} = \begin{bmatrix} p & \sqrt{pq}\prod_{k=1}^{m} s_k \\ \sqrt{pq}\prod_{k=1}^{m} s_k^* & q \end{bmatrix}.
\tag{S16}
$$

Thus, we can calculate $H_{\mathcal{S}}$, $H_{\mathcal{SF}}$, and $H_{\mathcal{S}\bar{\mathcal{F}}}$ efficiently because $\rho_{\mathcal{S}}$, $\rho_{\mathcal{SF}}$, and $\rho_{\mathcal{S}\bar{\mathcal{F}}}$ have been reduced to rank-two density matrices. In Eq. (S15), the basis set of $\rho_{\mathcal{SF}}$ is $\{|0_{\mathcal{S}}\rangle|\mathcal{F}_0\rangle, |1_{\mathcal{S}}\rangle|\mathcal{F}_1\rangle\}$, which is an orthogonal one due to the existence of pointer basis $\{|0_{\mathcal{S}}\rangle, |1_{\mathcal{S}}\rangle\}$. Note that the similar condition also holds for $\rho_{\mathcal{S}}$ and $\rho_{\mathcal{S}\bar{\mathcal{F}}}$ but not for $\rho_{\mathcal{F}}$, since $|\mathcal{F}_0\rangle$ and $|\mathcal{F}_1\rangle$ are not necessarily orthogonal [see Eq. (S11)], which makes the calculation of $H_{\mathcal{F}}$ not easy. Fortunately, $|\Psi^{\oslash}_{\mathcal{SE}}\rangle$ is a pure state by definition, thus, by the symmetry of the von Neumann entropy, we have $H_{\mathcal{F}} = H_{\mathcal{S}\bar{\mathcal{F}}}$.

Hence, $I(\mathcal{S}:\mathcal{F})$ can be computed exactly [11] regardless of the sizes of $\mathcal{E}$ and $\mathcal{F}$:

$$
I(\mathcal{S}:\mathcal{F}) = h(\lambda^{+}_{1,N,p}) + h(\lambda^{+}_{1,m,p}) - h(\lambda^{+}_{m+1,N,p}),
\tag{S17}
$$

where $h(x) = -x\log_2(x) - (1-x)\log_2(1-x)$, and $\lambda^{\pm}_{a,b,p}$ are the eigenvalues of the density matrices $\rho_{\mathcal{S}}, \rho_{\mathcal{S}\bar{\mathcal{F}}}$, and $\rho_{\mathcal{SF}}$, given by

$$
\lambda^{\pm}_{a,b,p} = \frac{1}{2}\left(1 \pm \sqrt{(2p-1)^2 + 4p(1-p)\Pi^{b}_{k=a}|s_k|^2}\right).
\tag{S18}
$$

To get more insight from the results above, we investigate $\rho_{\mathcal{S}}$, $\rho_{\mathcal{S}\bar{\mathcal{F}}}$, $\rho_{\mathcal{SF}}$ for a special case: $\theta^{j}_{k} = \theta^{j}, \phi^{0}_{k} - \phi^{1}_{k} = 0,$

in which they can be reduced to

$$\rho_{\mathcal{S}} = \begin{bmatrix} p & \sqrt{pq}\cos^{N}(\frac{\Delta\theta}{2}) \\ \sqrt{pq}\cos^{N}(\frac{\Delta\theta}{2}) & q \end{bmatrix}, \tag{S19}$$

$$\rho_{\mathcal{S}\bar{\mathcal{F}}} = \begin{bmatrix} p & \sqrt{pq}\cos^{m}(\frac{\Delta\theta}{2}) \\ \sqrt{pq}\cos^{m}(\frac{\Delta\theta}{2}) & q \end{bmatrix}, \tag{S20}$$

$$\rho_{\mathcal{S}\mathcal{F}} = \begin{bmatrix} p & \sqrt{pq}\cos^{N-m}(\frac{\Delta\theta}{2}) \\ \sqrt{pq}\cos^{N-m}(\frac{\Delta\theta}{2}) & q \end{bmatrix}, \tag{S21}$$

where $\Delta\theta = \theta^0 - \theta^1$. We can use Eqs. (S19) - (S21) to interpret the behavior of $I(\mathcal{S}:\mathcal{F})$ displayed in Figs. 1B, 1C and 2D of the main text. Assuming $\Delta\theta \neq 0$ and $p = q = 1/2$, in the large N limit, $H_{\mathcal{S}} \to 1$ since $\cos^N(\Delta\theta/2) \to 0$, indicating that $\mathcal{S}$ is fully decohered. In this case, $I(\mathcal{S}:\mathcal{F}) \approx 1 + H_{\mathcal{F}} - H_{\mathcal{S}\mathcal{F}}$. When $m \ll N$, the off-diagonal terms still exist for $\rho_{\mathcal{S}\bar{\mathcal{F}}}$ but vanish for $\rho_{\mathcal{S}\mathcal{F}}$, so $I(\mathcal{S}:\mathcal{F}) \approx 1 + H_{\mathcal{F}} - 1 = H_{\mathcal{F}}$. In this regime, the observer can only collect little information about $\mathcal{S}$ by measuring $\mathcal{F}$. As m increases, $H_{\mathcal{F}} \to 1$ rapidly, caused by the stronger decoherence of $\rho_{\mathcal{S}\bar{\mathcal{F}}}$, which indicates the onset of the plateau of $I(\mathcal{S}:\mathcal{F})$ and the emergent classical reality. Furthermore, as m grows close to N, $H_{\mathcal{S}\mathcal{F}}$ begins to decrease, leading to the mutual information $I(\mathcal{S}:\mathcal{F}) = 1 + 1 - H_{\mathcal{S}\mathcal{F}} \to 2$. In such a scenario, all the quantum information is stored in $\rho_{\mathcal{S}\mathcal{F}}$.

D. Local observables as quantifiers for quantum Darwinism

Now shifting focus to local observables, we show the full mathematical proof for how a chosen set of observables serves as a quantifier for classicality. First, we start with the limiting case of the singly-branching structures.

Singly-branching states of arbitrary dimension: In Ref. [28], the following theorem was derived:

Given a pure state $|\psi_{\mathcal{S}\mathcal{F}\bar{\mathcal{F}}}\rangle$ *such that* $\mathcal{D}(\mathcal{S}:\tilde{\mathcal{F}}) \leq \epsilon_{\mathcal{D}}$ *and* $|I(\mathcal{S}:\mathcal{F}) - H_{\mathcal{S}}| \leq \epsilon_I$*, then for all* $\epsilon_{\mathcal{D}}, \epsilon_I > 0$ *there exists* $\eta(\epsilon_{\mathcal{D}}, \epsilon_I) \geq 0$ *with* $\eta \in \mathcal{O}(\epsilon_{\mathcal{D}}, \epsilon_I)$ *and a branching state* $|\mathrm{GHZ}\rangle = \sum_{n=1}^{D_{\mathcal{S}}} \sqrt{y_n}|n\rangle|\mathcal{F}_n\rangle|\bar{\mathcal{F}}_n\rangle$ *such that*

$$|\langle\psi_{\mathcal{S}\mathcal{F}\bar{\mathcal{F}}}|\mathrm{GHZ}\rangle|^2 \geq 1 - \eta(\epsilon_{\mathcal{D}}, \epsilon_I). \tag{S22}$$

Essentially this postulates that the states that can support classicality are arbitrarily close to a GHZ state. Therefore, let's consider the following structure of states:

$$|\psi_{\mathcal{S}\mathcal{E}}\rangle = \sum_{n=1}^{D_{\mathcal{S}}} \sqrt{p_n}|n\rangle|\mathcal{F}_n\rangle|\bar{\mathcal{F}}_n\rangle, \tag{S23}$$

such that the states $|n\rangle$ form the pointer basis, and the states $|\bar{\mathcal{F}}\rangle$ form an orthonormal basis, while $|\mathcal{F}\rangle$ are not necessarily orthonormal. The physical assumption here is that the fragment $\mathcal{F}$ is small while the rest of the environment $\bar{\mathcal{F}}$ is large enough to ensure orthogonality. Consider now the local observable:

$$\mathcal{O} = A \otimes B \otimes \mathbb{I} \otimes ... \otimes \mathbb{I}. \tag{S24}$$

Taking the expectation value with consideration of the branching states leads to

$$\langle\psi_{\mathcal{S}\mathcal{E}}|\mathcal{O}|\psi_{\mathcal{S}\mathcal{E}}\rangle = \sum_{n=1}^{D_{\mathcal{S}}} p_n \langle n|A|n\rangle\langle\mathcal{F}_n|B|\mathcal{F}_n\rangle. \tag{S25}$$

This is a valid approximation since the rest of the environment is large. The above expectation value tends to zero if $\langle n|A|n\rangle = 0$. In fact, if we choose the observable A such that it rotates the pointer basis $A|n\rangle \to |n+1\rangle$; e.g. the operation that does this is σ_x if the pointer states are the eigenstates of σ_z: $|0\rangle$ and $|1\rangle$. With this choice of observable, we guarantee that whenever the wave function $|\psi_{\mathcal{S}\mathcal{E}}\rangle$ tends to a branching form, the expectation of $\mathcal{O}$ is arbitrarily close to 0. One immediate question one might ask is the following: *Why is this the minimal structure of local observables?* If we only consider the observable

$$\mathcal{O} = A \otimes \mathbb{I} \otimes ... \otimes \mathbb{I}, \tag{S26}$$

then

$$\langle\psi_{\mathcal{S}\mathcal{E}}|\mathcal{O}|\psi_{\mathcal{S}\mathcal{E}}\rangle \approx \sum_{n=1}^{D_{\mathcal{S}}} p_n \langle n|A|n\rangle. \tag{S27}$$

For equal probabilities, we get

$$\langle \psi_{\mathcal{SE}}|\mathcal{O}|\psi_{\mathcal{SE}}\rangle = (1/D_{\mathcal{S}})\sum_{n=1}^{D_{\mathcal{S}}}\langle n|A|n\rangle = (1/D_{\mathcal{S}})\mathrm{Tr}(A), \tag{S28}$$

hence, any traceless observable would lead to zero expectation value when the plateau forms. Therefore, the minimal structure needed is the following:

$$\mathcal{O} = A\otimes B\otimes \mathbb{I}\otimes ...\otimes \mathbb{I}. \tag{S29}$$

Where the operator B has support over a single qubit's Hilbert space. Now, we explicitly show the general form of the expectation value of the above local observables for arbitrary states $|\psi\rangle$. In particular, on the Hilbert space of system, fragment, and complementary fragment, we have,

$$\begin{aligned}|\psi\rangle &= \sum_{i,j,k}\sqrt{q_{i,j,k}}|i\rangle|F_j\rangle|\bar{\mathcal{F}}_k\rangle \\ &= \sum_{n,j}\sqrt{q_{n,j,n}}|n\rangle|F_j\rangle|\bar{\mathcal{F}}_n\rangle + \sum_{i\neq k,j}\sqrt{q_{i,j,k}}|i\rangle|F_j\rangle|\bar{\mathcal{F}}_k\rangle \\ &= \sqrt{1-r}\left(\sum_{n}\sqrt{p_n}|n\rangle|\mathcal{F}_n\rangle|\bar{\mathcal{F}}_n\rangle\right) + \sqrt{r}|\phi\rangle \\ &= \sqrt{1-r}|\psi_{\mathcal{SE}}\rangle + \sqrt{r}|\phi\rangle,\end{aligned} \tag{S30}$$

and then by definition $\langle\phi|\psi_{\mathcal{SE}}\rangle = 0$. From this general decomposition (such that $r\in[0,1]$) we see,

$$\langle\mathcal{O}\rangle = (1-r)\langle\psi_{\mathcal{SE}}|\mathcal{O}|\psi_{\mathcal{SE}}\rangle + r\langle\phi|\mathcal{O}|\phi\rangle + 2\sqrt{r(1-r)}\mathrm{Re}(\langle\psi_{\mathcal{SE}}|\mathcal{O}|\phi\rangle). \tag{S31}$$

Similar to before, if we choose the observable A such that $A|n\rangle\to|n+1\rangle$ we get,

$$\langle\mathcal{O}\rangle = r\langle\phi|\mathcal{O}|\phi\rangle + 2\sqrt{r(1-r)}\mathrm{Re}(\langle\psi_{\mathcal{SE}}|\mathcal{O}|\phi\rangle), \tag{S32}$$

which we can choose to be nonzero by picking the appropriate operator B. Hence, for the general case ($r\neq 0$), we have a nonzero expectation value. Only when we are close to a branching structure can we guarantee that $\langle\mathcal{O}\rangle$ is equal to zero. From the theorem derived in [28], the expectation value we are proposing not only captures the plateau in the mutual information but also captures 'epsilon' discord. Thus, if we observe that $\langle\mathcal{O}\rangle$ is at a zero plateau, we guarantee that the mutual information is δ within a plateau and discord is less or equal than ϵ, such that δ and ϵ are arbitrarily small as guaranteed by the theorem derived in Ref. [28].

Supplementary Text 2 - Experimental Information

A. Device information

Our experiments are performed on a superconducting quantum processor possessing 121 frequency-tunable transmon qubits and 220 tunable couplers [34], where we choose a 3×3 and a 3×4 two-dimensional sub-lattice to explore the robustness of quantum Darwinism (Fig. 3 of the main text) and the emergent branching structure (Fig. 2 of the main text), respectively. The typical performance for these qubits is shown in fig. S1. The median values of energy relaxation time T_1 and spin-echo dephasing time T_2^{SE} for 9-qubit (12-qubit) system are 141 μs (134 μs), and 18 μs (18 μs) respectively, as depicted in fig. S1A and B. For the 9-qubit (12-qubit) configuration, the median Pauli errors of single-qubit gate, two-qubit CZ gate, along with the median readout error are 0.033% (0.037%), 0.246% (0.246%) and 0.669% (0.662%), which are shown in fig. S1C and D. See Ref. [34, 35] for further details of the experimental setup.

B. Optimization of experimental circuit

The circuit shown in Fig. 2B of the main text is a conceptual representation of the 12-qubit model in Fig. 2A of the main text, which requires long-range connectivity between the qubits. However, our physical device is a rectangular

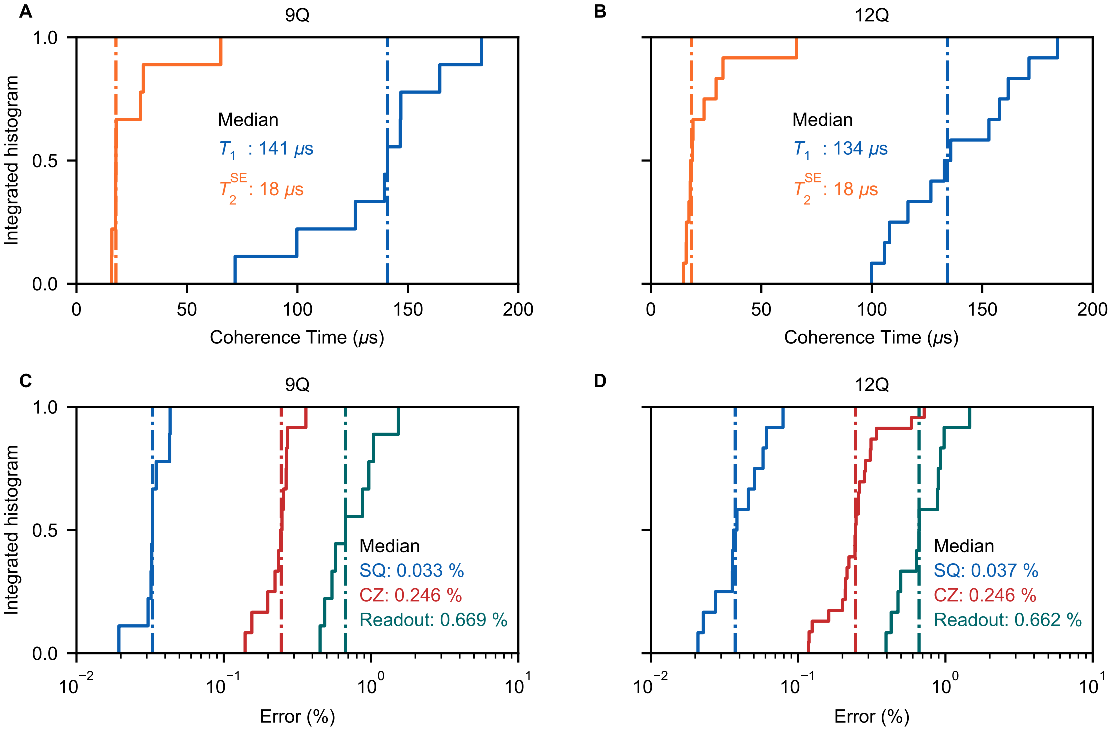

Figure S1. **Integrated histograms of typical qubit performance for 9-qubit and 12-qubit systems.** (**A-B**) Statistics of qubit energy relaxation time T_1 (blue lines) and spin-echo dephasing time T_2^{SE} (orange lines) for 9-qubit and 12-qubit systems. (**C-D**), Statistics of single-qubit gate error (SQ, blue lines), two-qubit CZ gate error (CZ, red lines), and readout error (green lines) for 9-qubit and 12-qubit systems. Gate errors are obtained by simultaneous cross-entropy benchmarking (XEB) [49]. The readout error is calculated as the mean value of $|0\rangle$ and $|1\rangle$ readout errors. The dashed-dotted lines indicate the median values of these parameters.

lattice (fig. S2A) with nearest-neighboring coupling, which cannot realize all the interactions between the system and environment directly. For example, as depicted in fig. S2B, the four corner qubits cannot interact with the central qubits directly. To overcome this, we utilize SWAP gates (orange lines) to bridges the interaction between the corner qubits and the system qubits. In our experiment, it is convenient to realize high-fidelity arbitrary single-qubit gates and CZ gates instead of random U_k conditional gates or SWAP gates. On the other hand, reducing the circuit depth and the number of gates is crucial to mitigate circuit errors. Therefore, we use Qiskit [50] to transpile the raw circuits into the desired ones which consist of single-qubit rotations and CZ gates.

C. Noisy simulation

In the main text, we perform the noisy simulation following the procedure described in Ref. [51]. There are various experimental errors, among which we mainly consider decoherence, depolarizing, and readout error (see table S1). More specifically, we use Qiskit's [50] noisy simulator to randomly sample the corresponding error operators and perform this process under different measurement bases for tomography. This process is similar to what is carried out experimentally. The related parameters and error rates listed in table S1 are also consistent with the experimental measurements. Note that $\epsilon_{\mathrm{SQ,idle}}$ is applied to those idle qubits when executing CZ gates in the same layer.

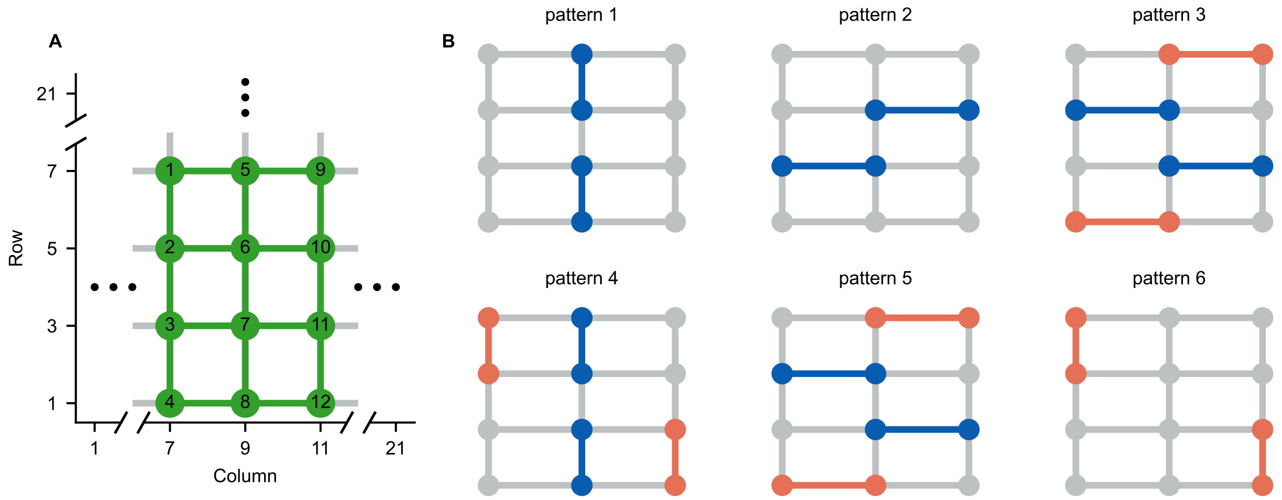

Figure S2. **The chip layout and two-qubit gate patterns used for observing emergent classical reality.** (**A**) The physical layout for the 12-qubit system. The circles denote qubits, and the lines represent couplers. The system consists of two qubits living in the center of the lattice (Q_6 and Q_7), surrounded by ten environment qubits. (**B**) Six two-qubit gate patterns corresponding to the circuit shown in Fig. 2B in the main text (the $U_{\text{tomo.}}$ is not displayed here). In each two-qubit gate pattern, two blue circles connected by a blue line depict the interaction between the system and the environment. Orange circles and lines represent SWAP gates. In the experiment, all two-qubit gates are decomposed into single-qubit rotations and CZ gates.

Table S1. The parameters and error rates used in the noisy simulation.

Parameters (mean) \ System	9-qubit	12-qubit
Single-qubit gate time t_{SQ}	20 ns	20 ns
Single-qubit idle gate time $t_{\text{SQ,idle}}$	47 ns	47 ns
CZ gate time t_{CZ}	47 ns	47 ns
Single-qubit gate error ϵ_{SQ}	0.033 %	0.043 %
Single-qubit idle gate error $\epsilon_{\text{SQ,idle}}$	0.082 %	0.079 %
CZ gate error ϵ_{CZ}	0.238 %	0.277 %
Readout error $\epsilon_{\text{readout}}$	0.8 %	0.8 %
T_1	135 μs	135 μs
T_ϕ	40 μs	40 μs
shots	5,000,000	1,000,000

REFERENCES AND NOTES

1. W. H. Zurek, Decoherence, einselection, and the quantum origins of the classical. *Rev. Mod. Phys.* **75**, 715–775 (2003).

2. M. Schlosshauer, *Decoherence and the Quantum-to-Classical Transition* (Springer, 2007).

3. E. Joos, H. D. Zeh, C. Kiefer, D. Giulini, J. Kupsch, I.-O. Stamatescu, *Decoherence and the Appearance of a Classical World in Quantum Theory* (Springer, 2003).

4. W. H. Zurek, Pointer basis of quantum apparatus: Into what mixture does the wave packet collapse? *Phys. Rev. D* **24**, 1516–1525 (1981).

5. W. H. Zurek, Environment-induced superselection rules. *Phys. Rev. D* **26**, 1862–1880 (1982).

6. W. H. Zurek, Einselection and decoherence from an information theory perspective. *Ann. Phys.* **512**, 855–864 (2000).

7. H. Ollivier, D. Poulin, W. H. Zurek, Objective properties from subjective quantum states: Environment as a witness. *Phys. Rev. Lett.* **93**, 220401 (2004).

8. H. Ollivier, D. Poulin, W. H. Zurek, Environment as a witness: Selective proliferation of information and emergence of objectivity in a quantum universe. *Phys. Rev. A* **72**, 042113 (2005).

9. W. H. Zurek, Quantum Darwinism. *Nat. Phys.* **5**, 181–188 (2009).

10. W. H. Zurek, Quantum theory of the classical: Einselection, envariance, quantum Darwinism and extantons. *Entropy* **24**, 1520 (2022).

11. A. Touil, B. Yan, D. Girolami, S. Deffner, W. H. Zurek, Eavesdropping on the decohering environment: Quantum darwinism, amplification, and the origin of objective classical reality. *Phys. Rev. Lett.* **128**, 010401 (2022).

12. G. L. Giorgi, F. Galve, R. Zambrini, Quantum Darwinism and non-Markovian dissipative dynamics from quantum phases of the spin-1/2 *XX* model. *Phys. Rev. A* **92**, 022105 (2015).

13. N. Balanesković, Random unitary evolution model of quantum Darwinism with pure decoherence. *Eur. Phys. J. D* **69**, 232 (2015).

14. N. Balaneskovic, M. Mendler, Dissipation, dephasing and quantum Darwinism in qubit systems with random unitary interactions. *Eur. Phys. J. D* **70**, 177 (2016).

15. P. A. Knott, T. Tufarelli, M. Piani, G. Adesso, Generic emergence of objectivity of observables in infinite dimensions. *Phys. Rev. Lett.* **121**, 160401 (2018).

16. N. Milazzo, S. Lorenzo, M. Paternostro, G. M. Palma, Role of information backflow in the emergence of quantum Darwinism. *Phys. Rev. A* **100**, 012101 (2019).

17. S. Campbell, B. Ç. Çakmak, O. E. Müstecaplioğlu, M. Paternostro, B. Vacchini, Collisional unfolding of quantum Darwinism. *Phys. Rev. A* **99**, 042103 (2019).

18. E. Ryan, M. Paternostro, S. Campbell, Quantum Darwinism in a structured spin environment. *Phys. Lett. A* **416**, 127675 (2021).

19. G. García-Pérez, D. A. Chisholm, M. A. C. Rossi, G. M. Palma, S. Maniscalco, Decoherence without entanglement and quantum Darwinism. *Phys. Rev. Res.* **2**, 012061(R) (2020).

20. S. Lorenzo, M. Paternostro, G. M. Palma, Anti-zeno-based dynamical control of the unfolding of quantum Darwinism. *Phys. Rev. Res.* **2**, 013164 (2020).

21. D. Girolami, A. Touil, B. Yan, S. Deffner, W. H. Zurek, Redundantly amplified information suppresses quantum correlations in many-body systems. *Phys. Rev. Lett.* **129**, 010401 (2022).

22. P. Duruisseau, A. Touil, S. Deffner, Pointer states and quantum Darwinism with two-body interactions. *Entropy* **25**, 1573 (2023).

23. W. H. Zurek, *Decoherence and Quantum Darwinism: From Quantum Foundations to Classical Reality* (Cambridge Univ. Press, 2025).

24. H. Ollivier, W. H. Zurek, Quantum discord: A measure of the quantumness of correlations. *Phys. Rev. Lett.* **88**, 017901 (2001).

25. F. Anza, J. P. Crutchfield, Geometric quantum thermodynamics. *Phys. Rev. E* **106**, 054102 (2022).

26. F. Anza, J. P. Crutchfield, Quantum information dimension and geometric entropy. *PRX Quantum* **3**, 020355 (2022).

27. F. Anza, J. P. Crutchfield, Beyond density matrices: Geometric quantum states. *Phys. Rev. A* **103**, 062218 (2021).

28. A. Touil, F. Anza, S. Deffner, J. P. Crutchfield, Branching states as the emergent structure of a quantum universe. *Quantum* **8**, 1494 (2024).

29. R. Blume-Kohout, W. H. Zurek, A simple example of "Quantum Darwinism": Redundant information storage in many-spin environments. *Found. Phys.* **35**, 1857–1876 (2005).

30. R. Blume-Kohout, W. H. Zurek, Quantum Darwinism: Entanglement, branches, and the emergent classicality of redundantly stored quantum information. *Phys. Rev. A* **73**, 062310 (2006).

31. M. A. Ciampini, G. Pinna, P. Mataloni, M. Paternostro, Experimental signature of quantum Darwinism in photonic cluster states. *Phys. Rev. A* **98**, 020101(R) (2018).

32. M.-C. Chen, H.-S. Zhong, Y. Li, D. Wu, X.-L. Wang, L. Li, N.-L. Liu, C.-Y. Lu, J.-W. Pan, Emergence of classical objectivity of quantum Darwinism in a photonic quantum simulator. *Sci. Bull.* **64**, 580–585 (2019).

33. T. K. Unden, D. Louzon, M. Zwolak, W. H. Zurek, F. Jelezko, Revealing the emergence of classicality using nitrogen-vacancy centers. *Phys. Rev. Lett.* **123**, 140402 (2019).

34. S. Xu, Z.-Z. Sun, K. Wang, L. Xiang, Z. Bao, Z. Zhu, F. Shen, Z. Song, P. Zhang, W. Ren, X. Zhang, H. Dong, J. Deng, J. Chen, Y. Wu, Z. Tan, Y. Gao, F. Jin, X. Zhu, C. Zhang, N. Wang, Y. Zou, J. Zhong, A. Zhang, W. Li, W. Jiang, L.-W. Yu, Y. Yao, Z. Wang, H. Li, Q. Guo, C. Song, H. Wang, D.-L. Deng, Digital simulation of projective non-abelian anyons with 68 superconducting qubits. *Chinese Phys. Lett.* **40**, 060301 (2023).

35. Z. Bao, S. Xu, Z. Song, K. Wang, L. Xiang, Z. Zhu, J. Chen, F. Jin, X. Zhu, Y. Gao, Y. Wu, C. Zhang, N. Wang, Y. Zou, Z. Tan, A. Zhang, Z. Cui, F. Shen, J. Zhong, T. Li, J. Deng, X. Zhang, H. Dong, P. Zhang, Y.-R. Liu, L. Zhao, J. Hao, H. Li, Z. Wang, C. Song, Q. Guo, B. Huang, H. Wang, Creating and controlling global Greenberger-Horne-Zeilinger entanglement on quantum processors. *Nat. Commun.* **15**, 8823 (2024).

36. I. Bengtsson, K. Zyczkowski, *Geometry of Quantum States: An Introduction to Quantum Entanglement* (Cambridge Univ. Press, 2009).

37. M. Zwolak, W. H. Zurek, Complementarity of quantum discord and classically accessible information. *Sci. Rep.* **3**, 1729 (2013).

38. A. Touil, S. Deffner, Information scrambling versus decoherence—Two competing sinks for entropy. *PRX Quantum* **2**, 010306 (2021).

39. A. Touil, S. Deffner, Information scrambling—A quantum thermodynamic perspective. *Europhys. Lett.* **146**, 48001 (2024).

40. W. H. Zurek, Quantum Darwinism, classical reality, and the randomness of quantum jumps. *Phys. Today* **67**, 44–50 (2014).

41. F. G. S. L. Brandão, M. Piani, P. Horodecki, Generic emergence of classical features in quantum Darwinism. *Nat. Commun.* **6**, 7908 (2015).

42. C. J. Riedel, W. H. Zurek, Quantum Darwinism in an everyday environment: Huge redundancy in scattered photons. *Phys. Rev. Lett.* **105**, 020404 (2010).

43. C. J. Riedel, W. H. Zurek, M. Zwolak, The rise and fall of redundancy in decoherence and quantum Darwinism. *New J. Phys.* **14**, 083010 (2012).

44. C. J. Riedel, W. H. Zurek, Redundant information from thermal illumination: Quantum Darwinism in scattered photons. *New J. Phys.* **13**, 073038 (2011).

45. W. H. Zurek, "Quantum Darwinism, decoherence, and the randomness of quantum jumps" (Tech. Rep. LA-UR-14-24063, Los Alamos National Laboratory, 2014).

46. K. Vogel, H. Risken, Determination of quasiprobability distributions in terms of probability distributions for the rotated quadrature phase. *Phys. Rev. A* **40**, 2847–2849 (1989).

47. W. H. Zurek, Preferred states, predictability, classicality and the environment-induced decoherence. *Prog. Theor. Phys.* **89**, 281–312 (1993).

48. W. H. Zurek, S. Habib, J. P. Paz, Coherent states via decoherence. *Phys. Rev. Lett.* **70**, 1187–1190 (1993).

49. S. Boixo, S. V. Isakov, V. N. Smelyanskiy, R. Babbush, N. Ding, Z. Jiang, M. J. Bremner, J. M. Martinis, H. Neven, Characterizing quantum supremacy in near-term devices. *Nat. Phys.* **14**, 595–600 (2018).

50. A. Javadi-Abhari, M. Treinish, K. Krsulich, C. J. Wood, J. Lishman, J. Gacon, S. Martiel, P. D. Nation, L. S. Bishop, A. W. Cross, B. R. Johnson, J. M. Gambetta, Quantum computing with Qiskit. arXiv:2405.08810 (2024).

51. L. Xiang, W. Jiang, Z. Bao, Z. Song, S. Xu, K. Wang, J. Chen, F. Jin, X. Zhu, Z. Zhu, F. Shen, N. Wang, C. Zhang, Y. Wu, Y. Zou, J. Zhong, Z. Cui, A. Zhang, Z. Tan, T. Li, Y. Gao, J. Deng, X. Zhang, H. Dong, P. Zhang, S. Jiang, W. Li, Z. Lu, Z.-Z. Sun, H. Li, Z. Wang, C. Song, Q. Guo, F. Liu, Z.-X. Gong, A. V. Gorshkov, N. Y. Yao, T. Iadecola, F. Machado, H. Wang, D.-L. Deng, Long-lived topological time-crystalline order on a quantum processor. *Nat. Commun.* **15**, 8963 (2024).
